# Electrical Impedance Tomography-Based Electronic Skin for Multi-Touch Tactile Sensing Using Hydrogel Material and FISTA Algorithm

**DOI:** 10.3390/s24185985

**Published:** 2024-09-15

**Authors:** Zhentao Jiang, Zhiyuan Xu, Mingfu Li, Hui Zeng, Fan Gong, Yuke Tang

**Affiliations:** 1 School of Mechanical Engineering and Mechanics, Xiangtan University, Xiangtan 411105, China; jiangzhentao_1017@163.com (Z.J.); mingfuli@xtu.edu.cn (M.L.);; 2 Key Laboratory of Nondestructive Testing of Ministry of Education, Nanchang Hangkong University, Nanchang 330063, China

**Keywords:** electronic skin, tactile sensor, multi-touch detection, image reconstruction, electrical impedance tomography

## Abstract

Flexible electronic skin (e-skin) can enable robots to have sensory forms similar to human skin, enhancing their ability to obtain more information from touch. The non-invasive nature of electrical impedance tomography (EIT) technology allows electrodes to be arranged only at the edges of the skin, ensuring the stretchability and elasticity of the skin’s interior. However, the image quality reconstructed by EIT technology has deteriorated in multi-touch identification, where it is challenging to clearly reflect the number of touchpoints and accurately size the touch areas. This paper proposed an EIT-based flexible tactile sensor that employs self-made hydrogel material as the primary sensing medium. The sensor’s structure, fabrication process, and tactile imaging principle were elaborated. To improve the quality of image reconstruction, the fast iterative shrinkage-thresholding algorithm (FISTA) was embedded into the EIDORS toolkit. The performances of the e-skin in aspects of assessing the touching area, quantitative force sensing and multi-touch identification were examined. Results showed that the mean intersection over union (MIoU) of the reconstructed images was improved up to 0.84, and the tactile position can be accurately imaged in the case of the number of the touchpoints up to seven (larger than two to four touchpoints in existing studies), proving that the combination of the proposed sensor and imaging algorithm has high sensitivity and accuracy in multi-touch tactile sensing. The presented e-skin shows potential promise for the application in complex human–robot interaction (HRI) environments, such as prosthetics and wearable devices.

## 1. Introduction

With the development of human–robot interaction (HRI) technology, traditional industrial robotic systems have evolved into collaborative robots (or cobots [1]) which allow for a physical interaction with humans in a shared workspace. The distance between humans and robots has been shortened, and there is an increasing need for robots to have the ability to engage in complex interactions with humans [1]. However, in the field of tactile sensing, current robotic electronic skins (e-skins) do not possess the sensory forms of human skin [2], making it challenging to achieve a safe, natural, and intuitive interaction between humans and robots. To enable such interactions in robotic systems, the e-skin field has emerged and become an important research topic in areas such as soft robotics and motion or physiological activity tracking over the past decade [3,4,5].

Flexible sensing technology is a research hotspot in the field of e-skin. Humans can perceive tactile stimuli, temperature differences, and pain because our skin is not only soft and deformable but also contains complex sensory receptors and neural networks [6]. A soft, stretchable, and elastic skin enhances tactile sensitivity, thus helping humans more accurately extract various information from tactile stimuli. In this context, skin deformation and tactile sensitivity are mutually coordinated and promoted [2]. However, traditional e-skins have difficulties meeting these requirements because they mostly use array sensors and wires composed of a large number of sensing units and circuits to collect signals. This scheme has two shortcomings. First, the elasticity of the skin is insufficient. Since the sensing units must be connected through wires, even with technologies like matrix wiring to reduce wires, the sensing area will still be filled with intersecting wires, thereby reducing the stretchability and deformability of the sensor. Second, the manufacturing process is cumbersome. The array structure complicates the manufacturing process and brings about issues of fragility and difficulty in repairs, especially in large-scale manufacturing [6,7]. A solution for eliminating the internal wires of the e-skin is to employ small RF devices integrated with two-dimensional signal transmission technology [8], but this will pose new problems in perceiving skin deformation and deployment in narrow areas. Another reported strategy is to combine visual sensors and tactile sensors, which are effective in sensing but require additional space for fixing the camera and keeping a necessary distance between the camera and the skin. Apparently, this method requires additional sensors and is restricted in scenarios where only a narrow space can be accessed. Therefore, the key to the e-skin is to manufacture flexible tactile sensors with an independent simple structure and good elasticity.

In recent years, electrical impedance tomography (EIT) technology has gained increasing attention in the field of robotic e-skins because it can provide continuous, real-time, large-area sensing with minimal wiring [9]. EIT technology is a non-destructive testing technique that attaches a series of electrodes to the surface of the object being measured, injecting a small current into the object’s interior. By measuring the voltage distribution, the internal impedance distribution of different regions of the object is calculated through an inversion algorithm to generate an image. The non-invasive nature of this technology eliminates the need for internal wiring within the skin, ensuring the simplicity and coherence of the skin structure. The real-time data acquisition and rapid response characteristics of EIT technology also provide a foundation for real-time tactile sensing [10]. EIT technology was primarily used in medical monitoring devices in the past, such as lung impedance imaging [11] and breast impedance detection technology [12]. In 2007, Kato and Nagakubo [2] first introduced EIT technology into the robotics field, and their sensor was made of conductive rubber. Since then, e-skin combined with EIT technology has received widespread attention. For instance, e-skins made using conductive fabrics have excellent stretching properties, can cover larger surfaces, and have good sensing effects even in the human elbow area [13,14]. Additionally, there exists e-skin made from ionic liquids and elastic films, with the films typically composed of latex or silicone; this fluid-based skin has excellent elasticity and self-healing properties [15,16] and can detect the dynamic properties of touchpoints. E-skin made from hydrogel materials possesses many promising features, including the ability to recover from large physical injuries while maintaining its original sensing properties [6], the capability to determine the location and type of dynamic haptic stimuli from vibrations generated by touch [17], and the sensitivity to detect even smaller force contacts through its unique sensing morphology [18]. Compared to traditional technologies, e-skin manufactured using EIT technology optimizes sensors’ complex structure and significantly reduces production costs. Existing research indicates that e-skin for robots must meet several requirements. First, it should be large, flexible, stretchable, and capable of covering various three-dimensional surfaces of the robots. Second, its fabrication should be simple, low-cost, reproducible, scalable, and durable. Thirdly, all hardware designs should be suitable for human activity environments [19]. Since EIT technology has already been used in medical imaging and possesses advantages such as non-invasiveness, simplicity, and low production costs, it holds promising research prospects in the field of e-skin.

The main concerns of the current research on EIT-based e-skin focus on the selection of skin materials and reconstruction algorithms. Since EIT technology relies on injecting a current, measuring voltage, and inversely determining the conductivity distribution, the skin material must be conductive. Moreover, the EIT image reconstruction process essentially involves solving a highly ill-posed problem, resulting in imprecise and non-unique solutions [20]. This leads to significant image artifacts and limited recognition capabilities, especially in multi-touch identification. To address these issues, this paper proposes a hydrogel-based flexible sensor. First, we briefly introduce the principles of EIT image reconstruction along with the hardware and sensor structure. Then, we compare the image quality of the proposed sensor processed by different algorithms and explore the relationship between conductivity changes and pressure variations. Finally, we present the reconstructed images of the proposed sensor under multi-touch conditions. The experiments utilized MATLAB 2023b software and the EIDORS 3.11 [21] toolkit for data-solving and image reconstruction. Compared to previous studies, the flexible sensor proposed in this paper demonstrates advantages in the number of identifiable targets and the quality of reconstructed images.

## 2. Materials and Methods

### 2.1. Electrical Impedance Tomography

**Figure 1:**
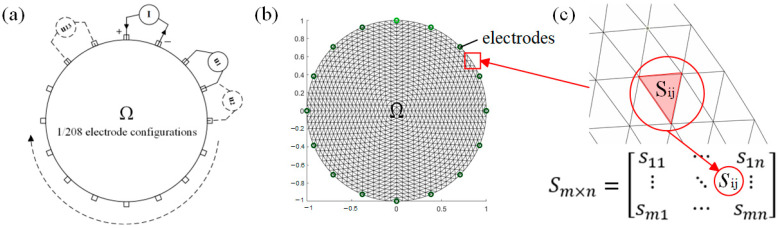
The working mechanism of EIT. (**a**) The basic working principle of the 16-electrode EIT system. (**b**) Discretizing the domain of the sensing material into a collection of a finite number of elements and nodes using the finite element method. (**c**) Sensitivity matrix composed of discrete grid cells.

EIT technology is based on Maxwell’s electromagnetic field theory, examining the relationship between the conductivity distribution within a sensitive field under specific excitation conditions and the boundary voltage measurements. In e-skin applications, EIT operates by uniformly arranging electrodes along the boundary of the skin and injecting a current in an excitation-specific pattern, thereby forming an electric field within the skin. The voltage data collected from each boundary electrode can be used to reconstruct the potential distribution within the skin. Figure 1a illustrates the working principle of a 16-electrode EIT system operating in adjacent excitation mode. In adjacent excitation mode, two electrodes are selected as the excitation electrodes in a clockwise manner each time, while all other electrodes (except adjacent ones) are selected as measurement electrodes. Considering all 16 current injection modes, namely (1,2), (2,3), …, (15,16), and (16,1), this process is repeated multiple times, resulting in the collection of 16 × 13 = 208 voltage data points. The collected data serve as input for the algorithm used to reconstruct the changes in internal conductivity. In the field Ω, the EIT forward problem can be expressed in the form of an elliptic partial differential equation (PDE) [22], expressed as

(1) $$\nabla \cdot \left(\sigma \cdot \nabla U\right)=0\;\mathrm{in}\;\Omega$$

The boundary conditions of Equation (1) are

(2) $$\nabla \sigma \cdot \frac{\partial U}{\partial \overset{⃑}{n}}=J\;on\;\partial \Omega$$

(3) $$U=U_{0}\;on\;\partial \Omega$$

where ∂Ω is the boundary of the sensitivity field, σ denotes the pointwise conductivity distribution of the medium, J denotes the external flux of current at the boundary, U denotes the internal potential of the sensitivity field, $U_{0}$ denotes the boundary potential, and $\overset{⃑}{n}$ is a unit normal vector pointing toward the exterior of the boundary. The physical meaning of the PDE and its boundary conditions is that the net current flowing into and out of any unique point within Ω is zero, and the boundary potential distribution of the sensitive field satisfies both the Neumann boundary condition and the Dirichlet boundary condition [22].

The forward problem is solved using the finite element method (FEM). Figure 1b illustrates the finite element mesh division within the Ω. In the EIT system, the relationship between the conductivity distribution σ and the boundary voltage measurements U is nonlinear.

(4) $$U=F\left(\sigma \right)\;$$

After dissecting the sensitive field into the pixel blocks according to the perturbation theory and discretizing it, Equation (4) can be expressed as

(5) $$U_{m\times 1}=S_{m\times n}\cdot g_{n\times 1}$$

where n is the number of discretized cells, m is the number of voltage measurements in one frame, $g_{n\times 1}$ is the conductivity distribution of all grid cells in the sensitive field, $U_{m\times 1}$ is the vector of normalized boundary measurements, and $S_{m\times n}$ is referred to as the sensitivity matrix (or Jacobian), representing the changing relationship between boundary voltage measurements and the conductivity distribution. The sensitivity of the *k*th element within the Ω can be calculated in Equation (6):

(6) $$S_{ij}\left(k\right)=\frac{\partial U_{ij}}{\partial \sigma_{k}}=\int_{\Omega_{k}}\nabla u\left(I_{i}\right)\cdot \nabla u\left(I_{j}\right)\;dV$$

Figure 1c represents the sensitivity matrix consisting of all grid cells within the Ω, and $\nabla u\left(I_{i}\right)$ and $\nabla u\left(I_{j}\right)$ denote the current gradient at the ith excitation and the jth acquisition.

**Figure 2:**
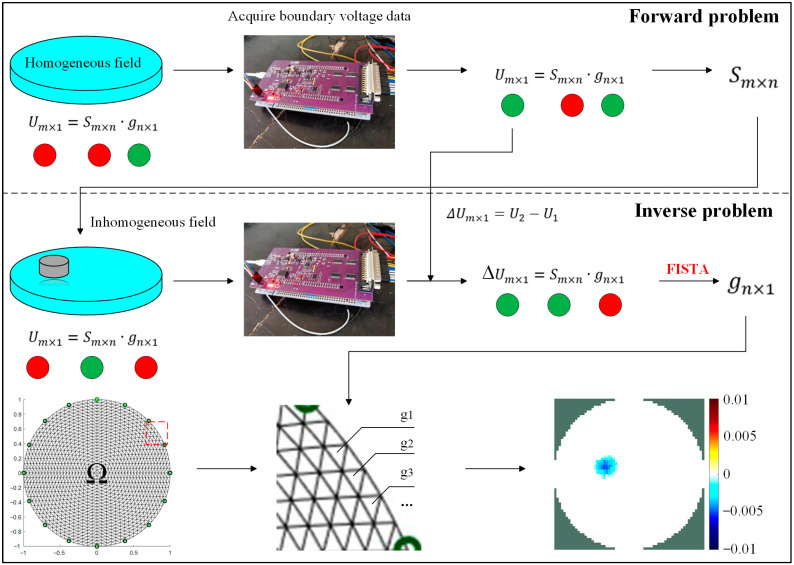
Imaging process of EIT-based tactile sensor. $U_{m\times 1}$ represents boundary voltage data, $S_{m\times n}$ represents the sensitivity matrix, and $g_{n\times 1}$ represents the conductivity distribution of all grid cells in Ω.

The EIT inverse problem is also known as the image reconstruction problem. It inverts the conductivity distribution $g_{n\times 1}$ in the sensitive field using the boundary voltage data $U_{m\times 1}$ through a sensitivity matrix when the potential distribution in the sensitive field is unknown. The working principle of the system is shown in Figure 2. Since the number of grid cells into which the region Ω is divided after finite element processing is usually much larger than the number of voltage data collected (n >> m); the inverse problem is a serious nonlinear and ill-posed problem, resulting in the low spatial resolution of reconstructed images. Currently, the core challenge in solving the EIT inverse problem is finding a suitable image reconstruction algorithm to obtain accurate images depicting conductivity parameters and boundary features. In the field of e-skin, existing research focuses on deep learning, such as EIT–CNNs (convolutional neural networks) and other deep learning algorithms [17,23] which are able to improve the quality of image reconstruction but require the support of large datasets. However, e-skin typically operates in resource-constrained environments (such as embedded systems), limiting the complexity and computation of deep learning models. Consequently, deep learning algorithms can hardly guarantee their original computational speed and accuracy in low-power, resource-constrained systems. In summary, algorithms with simpler models and lower hardware resource requirements should be considered in the field of e-skin.

The fast iterative shrinkage-thresholding algorithm (FISTA) is an efficient algorithm for solving sparse optimization problems, particularly convex optimization problems involving L_1_ regularization terms, such as the EIT reconstruction problem, which can be described as a least absolute shrinkage and selection operator (LASSO) model based on the sparse reconstruction method [24]. To improve the quality of image reconstruction, the FISTA was embedded into the EIDORS toolkit. In this model, the inputs to the algorithm are ${∆U}_{m\times 1}$ and $S_{m\times n}$ and the output of the algorithm is $g_{n\times 1}$. The system employs dynamic imaging to minimize errors caused by hardware and environmental factors. Compared to deep learning algorithms, the FISTA model is simpler, does not require excessive parameter adjustments, and usually forms a stable solution in fewer than 30 iterations [25], which can improve image reconstruction quality with fewer hardware resources.

### 2.2. Material Characterization and Sensor Fabrication

**Figure 3:**
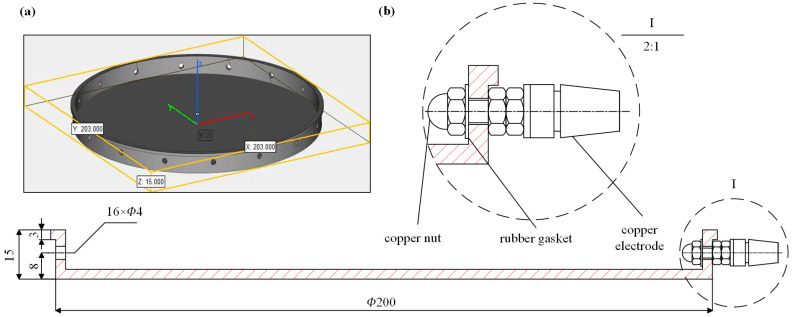
CAD drawing of the sensor container. (**a**) Resin housing and (**b**) copper electrodes assembled at the boundary of the housing. Dimensions are given in mm.

The proposed EIT-based tactile sensor consists of three parts, a skin container, 16 electrodes, and hydrogel-based skin. Figure 3 shows the structure and dimensions of the fabricated skin container, with all dimensions given in millimeters (mm). The circular container was made using 3D printing using resin materials (density: 1.3 g/cm^3^) and uniformly equipped with 16 through-holes, each with a diameter of 4 mm, along the container’s edge. The electrodes were assembled using M4 connecting posts and copper nuts, as shown in Figure 3b. The copper nuts served as the actual electrodes of the sensor, and the copper connecting posts transmitted the current released by the hardware through wires to the nuts. The elasticity of the rubber washer ensured a tight fit between the nut and the rim of the container, providing a tight seal.

**Figure 4:**
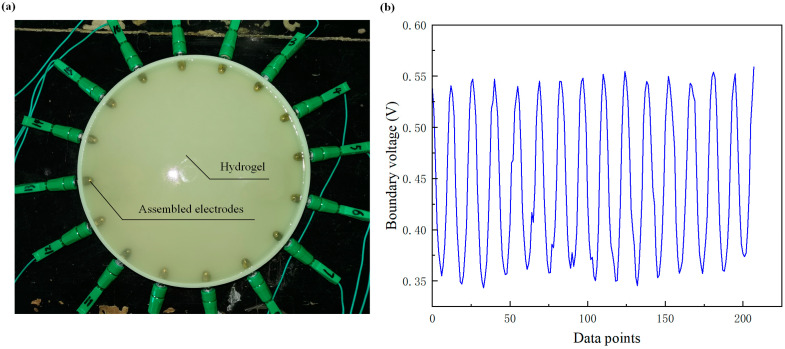
(**a**) The proposed EIT-based flexible sensor. (**b**) The waveform of boundary voltage in the case of a homogeneous field.

The proposed flexible sensor uses conductive hydrogel as the skin material. Hydrogel-based skin not only possesses good elasticity and biocompatibility but also has a high water content. This characteristic is conducive to forming a stable electric field within the skin, thus improving the accuracy of obtaining boundary voltage data. The hydrogel was made from agar, glycerol, and purified water. First, pour 300 g of purified water into a beaker and boil it. Then, add 5 g of agar powder (gel strength: (15 g/L, 20 °C)/(g/cm^2^) ≥ 400) and 100 g of glycerin, and stir the mixture evenly. Next, let the mixture cool to around 50 °C before pouring it into the skin container. Finally, allow it to cool at room temperature (20 °C) for 1 h to allow gel formation. The glycerin helps maintain the stability of the hydrogel over a longer period. The obtained hydrogel had a thickness of around 12 mm and a diameter of 194 mm. It could withstand a maximum pressure of about 5.41 kPa, with an elastic modulus of approximately 43.2 kPa. The appearance of the EIT-based flexible sensor is shown in Figure 4a. To verify the uniformity of the contact resistance of the 16 electrodes, the sensor was examined by a one-round sampling of the boundary voltages (208 datasets in the adjacent excitation mode) in the case of a homogeneous field, as shown in Figure 4b. It is observed that the 16 peaks and valleys of the waveform are uniform in general. This means that the differences of the distance and resistance between adjacent electrodes are retained on a small level, thereby validating the accuracy of the fabrication process of the sensor and guaranteeing a perceptible response in the case of an anomalous field.

**Figure 5:**
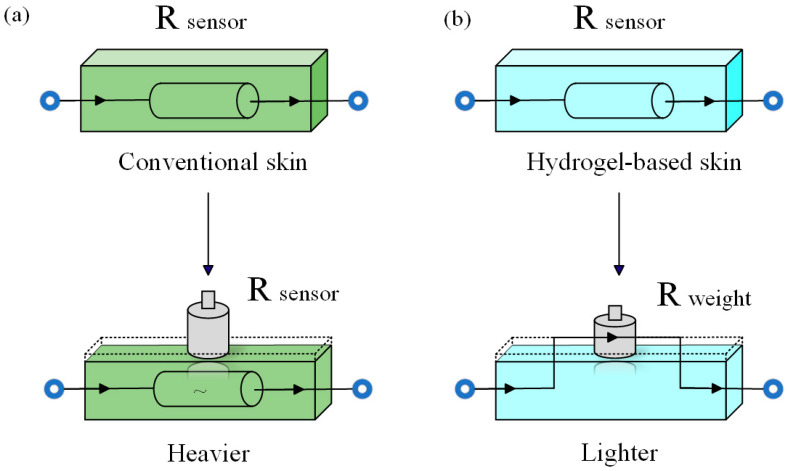
The touch mechanism of an EIT-based tactile sensor. (**a**) The conventional touch-detection mechanism. Physical compression causes material deformation, which leads to a change in the electrical resistance. (**b**) The hydrogel-based sensor touch-detection mechanism. Both the touch of highly conductive materials and the compression caused by pressure result in electrical resistance changes, which makes the hydrogel-based skin more sensitive to conductive changes.

Unlike EIT pressure sensors made from conductive rubber, conductive fabrics, and ion liquids with membrane materials, the proposed hydrogel tactile sensor can not only detect relative pressure changes but also improve the accuracy of touchpoint image reconstruction through electrical contact between the skin and conductive objects (such as human skin and metals), especially in the presence of multiple touchpoints. The principle of the conventional EIT-based sensor detecting relative pressure is similar to the piezoresistive effect of pressure-sensitive materials. When a point on the skin surface is subjected to pressure, the skin around the force point deforms due to the pressure, causing a change in local impedance and converting the object’s pressure into a change in the skin’s internal conductivity, as shown in Figure 5a. However, the touch-detection mechanism of our proposed sensor is different from the conventional kind. Since the electrical conductivity of the self-made hydrogel skin (less than 0.01 S/m) is much lower than that of human skin (approximately 0.09 S/m, susceptible to environmental influences) and most metallic contact objects, a local short-circuit phenomenon will occur when the contact object forms an electrical contact with the skin [16], as illustrated in Figure 5b. This characteristic makes the hydrogel skin more sensitive to conductive contact objects, allowing for more explicit images without applying a significant force. This feature is crucial in multi-touch identification for EIT sensors, as it effectively reduces the overall deformation of the skin when multiple force points are present, ensuring that each local force point’s relative position does not significantly shift on the skin and maintaining the stability of the internal electric field, thereby improving image reconstruction quality. However, the short-circuit approach cannot detect touches applied on non-conductive materials (such as plastic and rubber) because these materials do not cause significant changes in the electric field within the sensing area when in contact [16]. Although conductive fabrics and rubbers have a stable structure, they do not have the perceptual morphology of hydrogel skin. Therefore, they require more pressure to change the electrical resistance within the sensitive field. Though having better sensitivity and dynamic performance under a single contact point pressure, e-skins made from ion-liquids and membrane materials suffer significant deformation of the membrane material (such as latex and silicone) under multiple pressure points, resulting in subpar image quality for multi-touch identification. In conclusion, the proposed hydrogel-based skin aids EIT-based tactile sensors in achieving better multi-touch identification performance.

### 2.3. Sensing System

In order to obtain more information from impedance data in biomedical or industrial testing, EIT systems usually use an AC signal with a certain frequency and amplitude to obtain the entire impedance of the object under the test, including the resistive component (the real part of the impedance) and the capacitive component (the imaginary part of the impedance). Because the AC signal can effectively eliminate the effect of touch resistance caused by the frequent switching of electrodes during electrode rotation, the hardware can realize accurate impedance measurement. However, in the field of e-skin, people tend to pay more attention to the hardware cost and the system’s real-time performance and obtaining accurate impedance data will bring an additional burden to the hardware design and data processing process. Instead, DC is not only easy to use but also simplifies the analysis results. This design uses a DC power supply to power the hardware for the above reasons. The hardware consisted of the microcontroller STM32 and a separate PCB board structured in an upper and lower layer. The STM32 Ncleo-144 (MB1137) board in the lower layer was used to realize the data transfer with the computer and was combined with the upper PCB via pin headers in order to control the work of the modules on the upper PCB via the STM32 core.

**Figure 6:**
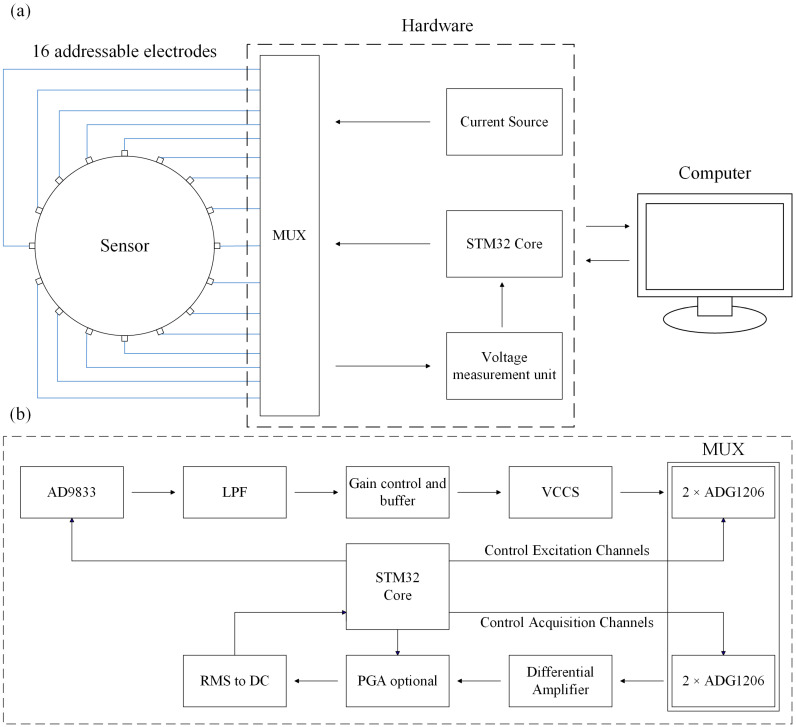
Block diagrams of the (**a**) EIT system and (**b**) main components of the hardware.

In general, the EIT system actually consists of the following components: sensors, hardware, and a computer, which are responsible for information extraction, transmission, and processing, respectively. The schematic diagram of the system is shown in Figure 6. During the experiment, we used the dual DC-regulated power supply to power the hardware. In the signal generation circuit, we generated a sinusoidal excitation signal with a frequency of 50 kHz through AD9833 and injected a current with an amplitude of 1 mA into the skin interior through the signal amplification circuit and the V/I module, which is a common excitation amplitude for EIT technology in medical imaging. In the data acquisition circuit, we integrated four 16:1 ADG1206 multiplexers to realize the selection of excitation and acquisition channels. In the mode of adjacent excitation, the impedance values between all electrode pairs were measured continuously, and 208 boundary voltage data can be acquired per frame. The hardware realized communication with the computer through the USB serial port, and the computer used MATLAB (MathWorks Inc., Natick, MA, USA) software and the EIDORS toolkit to realize the feedback voltage data-solving and perform the image reconstruction.

## 3. Experimental Evaluation

To demonstrate the effectiveness of the proposed sensor, we designed a series of experiments in this section to test its performance, and the results were analyzed. The experiments were implemented through the programming of MATLAB 2023b software and the EIDORS toolkit. First, we compared the quality of reconstructed images of EIT-based tactile sensors at single, double, and triple touchpoints using the FISTA and other different algorithms. The superior imaging results of the FISTA are crucial for improving the recognition accuracy of the sensors. Second, we performed quantitative force analysis at different touchpoints in hydrogel-based skin and verified that the sensor can sense relative changes in pressure by reconstructing images and conductivity change curves. Finally, tests for multi-touch were carried out, proving the sensor has higher sensitivity and accuracy in multi-touch identification.

### 3.1. Touch Detection Using Different Image Reconstruction Algorithms

To evaluate and compare the performances of various algorithms in the proposed EIT-based tactile sensor, in this section, we respectively reconstructed the images for the cases of single, double, and triple touchpoints in the actual working environment of the sensor, and the results of reconstructed images obtained by the FISTA were compared with those obtained by common GN, CG, and NOSER algorithms. The weights used in the experiment were identical in weight and size, and we kept all algorithms’ parameters constant during the experiment.

**Figure 7:**
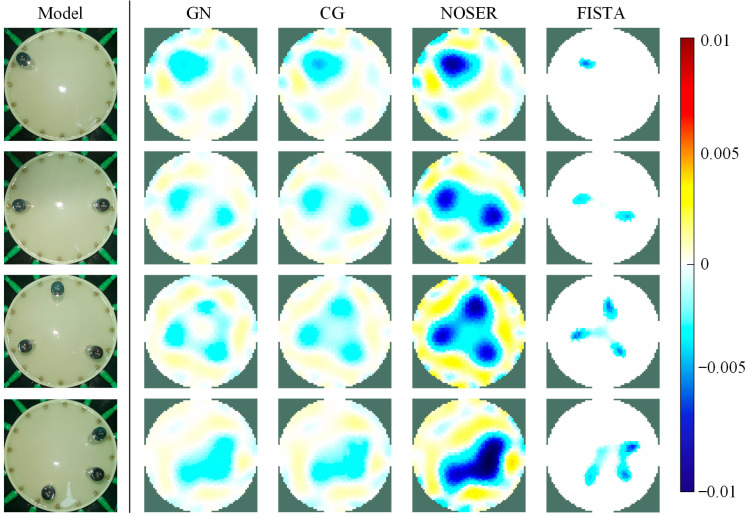
Imaging results of single- and multi-touch detection with different algorithms on EIT-based tactile sensor.

**Figure 8:**
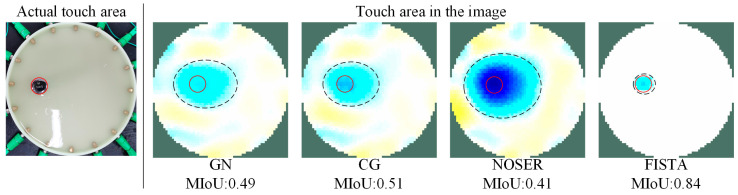
MIoU values for image reconstruction using the proposed method (FISTA) and other traditional methods.

The imaging results are shown in Figure 7. From the results, we can see that the reconstructed image’s darker color areas strongly correlate with the location of the pressure touchpoints, which indicates that the proposed sensor has the ability to detect the position of the pressure on the skin. However, different algorithms cause the final image recognition accuracy of the same model to vary greatly. To further demonstrate that the proposed algorithm can achieve more accurate touch area detection, the metric of the mean intersection over union (MIoU) was employed. The MIoU measures the difference between the actual touch area and the impedance distribution area in the reconstructed image [23]. A larger MIoU value indicates a greater overlap between the reconstructed image and the actual touch area, reflecting better image reconstruction accuracy.

(7) $$\mathrm{MIoU}=\frac{1}{k+1}\sum_{i=0}^{k}\frac{p_{ii}}{\sum_{j=0}^{k}p_{ij}+\sum_{j=0}^{k}p_{ij}-p_{ii}}$$

where k is the number of classes and $p_{ij}$ is the amount of pixels of class i inferred to belong to class j. The experimental results are shown in Figure 8. The MIoU values for other algorithms range from approximately 0.4 to 0.5, while the MIoU value after using the FISTA increased to 0.84. This indicates that the FISTA can effectively reduce the area of artifacts in the reconstructed image and define clearer contact boundaries, leading to more accurate conductivity visualization. This result is particularly important for the high-precision positioning in more complex touch scenarios. To summarize, this experiment proves that the FISTA helps the EIT-based tactile sensor to achieve better imaging quality.

### 3.2. Quantitative Force Imaging with Different Touchpoints

**Figure 9:**
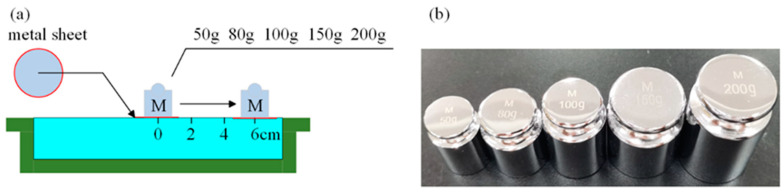
(**a**) Diagram of the position-moving weights at different touchpoints. A metal sheet is placed under the weights to maintain the same area of force. (**b**) Photo of the weights of different masses.

**Figure 10:**
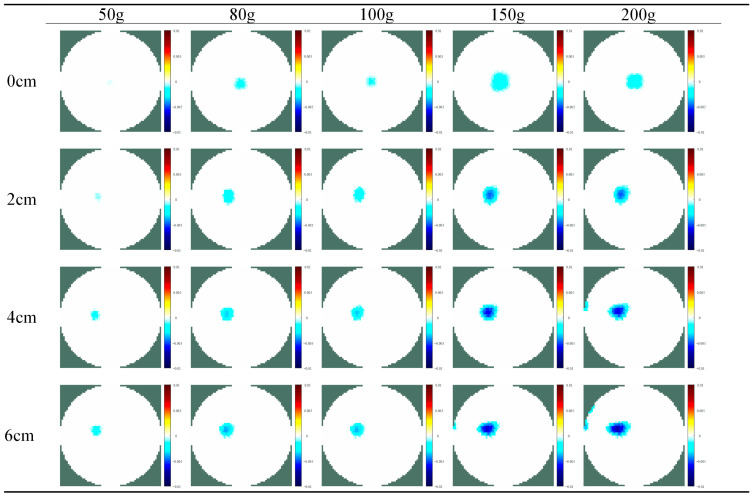
Reconstructed images of different masses of the weights applied on the hydrogel-based skin.

**Figure 11:**
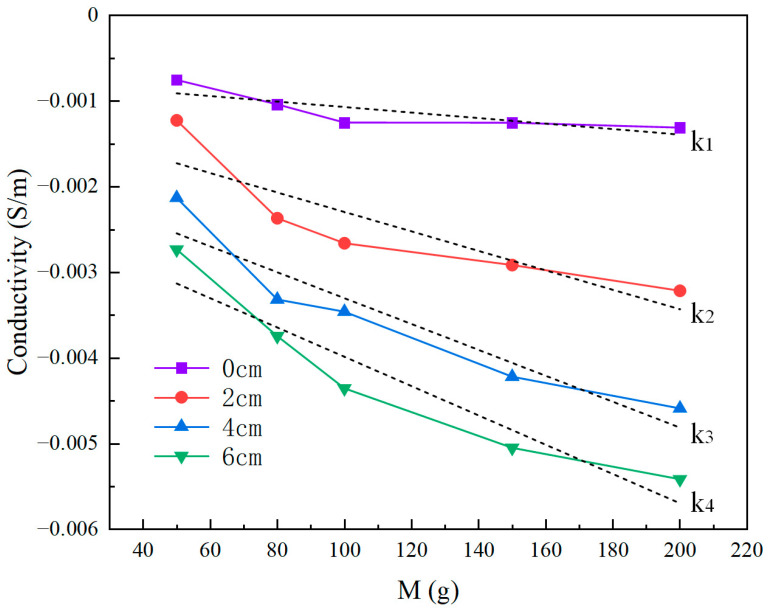
Relationship between the magnitude of weights and relative change in conductivity at different touchpoints. The slope of the fitted lines through data points indicates the relative magnitude of the sensitivity in different area (k_1_ < k_2_ < k_3_ < k_4_).

In some application scenarios (such as bionic prosthetics and social robots), to enhance the HRI experience, flexible tactile sensors need to recognize the touchpoint’s position and quantify the relative magnitude of the force applied to the point within a certain range. In order to know the accuracy of measurement in different areas over the whole e-skin surface, we have tested the proposed hydrogel-based skin with single-point touch in different areas, and Figure 9 shows the procedure of the experiment. All weights used in the experiment were made of metal with masses of 50 g, 80 g, 100 g, 150 g, and 200 g. First, a piece of metal sheet with negligible weight was placed on the skin’s surface, and all weights were placed on the piece of metal during the experiment to create a single pressure point with a constant area. Next, four different test points were divided at equal distances from the center to the boundary of the skin, and each test point was experimented on with five weights of different masses. During the experiment, the changes in the conductivity image caused by the weights of different masses and the absolute maximum of the conductivity change in the sensitive field were recorded. Several experiments were performed, and Figure 10 and Figure 11 show the reconstructed images and conductivity change curves at the end of the experiment. From the experimental results, it can be seen that in the same touch position and a certain pressure range, the conductivity and the load are approximately linear. The closer the position of the test point is to the electrode, the more obvious this variation becomes. This is due to the electric field formed by the current released by the electrode being naturally attenuated with the distance, which causes the sensitivity of the sensor force detection to increase from the center area to the boundary gradually. As the pressure position gets closer to the sensor’s center, the slope of the fitted lines through data points becomes smaller, implying that the pressure-induced conductivity change at different electric field strengths is limited. In short, these results indicate that the proposed sensor can be used to recognize the relative magnitude of pressure within a certain range.

### 3.3. Imaging of Multi-Touch

**Figure 12:**
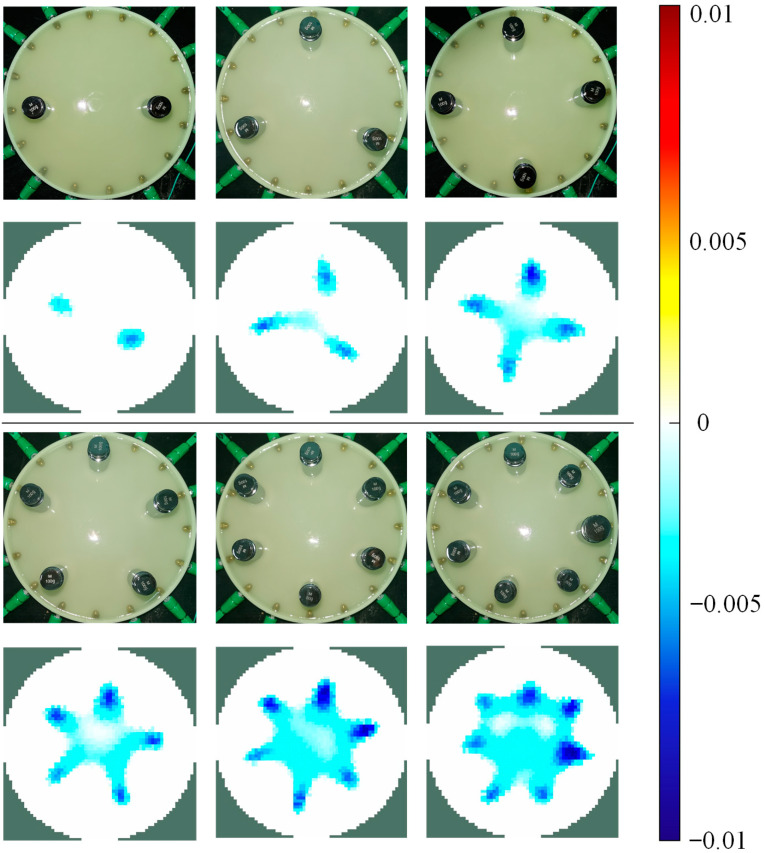
Weights applied onto the equally spaced 2 to 7 touchpoints of the sensor and the corresponding reconstructed images.

The capability of multi-touch identification directly impacts the performance of the advanced tactile sensing devices, for example, wearable devices to achieve gesture recognition [26] or soft robots to decipher multimodal stimulation using deep learning [27]. In this section, the experiments focused on the maximum number of touchpoints the sensor could identify through reconstructed images. For this purpose, we used multiple weights placed incrementally starting from the boundary position with the highest sensor sensitivity (6 mm, as shown in Figure 8) to simulate the situation where the sensor is subjected to an increasing number of touchpoints. The results are shown in Figure 12.

In existing studies, most EIT-based tactile sensors can usually identify a maximum of two to four touchpoints. For these sensors, a four-point touch is considered acceptable [28]. However, the proposed hydrogel sensor identified seven different touchpoints without any image post-processing, significantly enhancing the maximum number of touchpoints that EIT-based tactile sensors can reflect through images during multi-touch. Compared to previous research, the proposed sensor exhibited smaller artifact areas and clearer size contours. These results proved the effectiveness of hydrogel-based skin and the FISTA used in multi-touch identification. Nevertheless, the issue of image artifacts still became severe as the number of touchpoints increased, leading to poor imaging quality when identifying eight or more touchpoints. Due to hardware limitations, there is still significant room for improvement in this area. In summary, the proposed scheme enhances the ability of EIT-based tactile sensors to reflect multiple touchpoints through the reconstructed images.

Based on the above results, it can be concluded that the proposed sensor offers three main advantages over other soft tactile sensors. First, the sensor has a simple structure, without wiring within the sensing area. It uses only 16 electrodes arranged at the boundaries to create an electric field that can detect the conductive objects within the skin area of approximately 300 cm^2^, enabling the functionality of large-area sensing. Second, the sensor is easy to maintain. The hydrogel can be easily repaired using an adhesive, even after partial tearing or compression. The repaired e-skin retains its original sensing performance [17]. Third, the sensor has low power consumption. The current released by the electrodes is only 1 mA, allowing the entire system to be powered by a micro motor and facilitating the system miniaturization. In addition, hydrogel material is a biocompatible and highly elastic material, and thus can provide protective function for the robotic skin. This makes the sensor highly valuable for applications in bionic robots, social robots, prosthetics, and other devices that require close interaction with humans [17].

Compared to the current EIT-based sensors, the proposed system offers higher tactile recognition accuracy and improved multi-touch identification capabilities. In Figure 7 and Figure 8, we demonstrated that the proposed sensor could achieve more accurate detection of the touch area, even though the reconstruction was not processed by any image post-processing methods. Then, in Figure 10 and Figure 11, we illustrated the sensor’s effectiveness in detecting relative pressure across different areas of the skin. The sensor can detect pressures as low as 50 g across the entire sensing area and can reflect the pressure exerted by weights ranging from 50 to 200 g in various positions via conductivity images. Meanwhile, Figure 12 proved the sensor’s capability of identifying up to seven different touchpoints, which surpasses the maximum recognition numbers of existing EIT-based tactile sensors. Table 1 summarizes the comparisons.

The ability to recognize multiple touchpoints is particularly important for achieving more complex HRIs in applications such as wearable devices for gesture recognition [26] or soft robots that use deep learning to decode multimodal stimuli [27]. For the proposed sensor, it is expected to be accommodated into wearable devices by using microelectrodes and optimizing the power supply. For instance, the ion diode-based generators that can harvest mechanical energy from low-frequency human movements to generate electricity continuously [29] could be a potential solution.

## 4. Conclusions

In order to improve the performance of multi-touch identification and the accuracy of image reconstruction of the EIT-based tactile sensor, an EIT-based sensor taking advantage of a hydrogel and integrated with the FISTA for solving the nonlinear inverse problem was proposed in this work. The use of hydrogel material made the sensor capable of sensing a subtle change in force and tolerating a large deformation in the case of multi-point touching. The FISTA, which was embedded into the EIDORS package, reduced the area of artifacts and enhanced the resolution of the touch location in the reconstructed image. By using the metric of MIoU, the performances of the sensor for force quantification and multi-touch identification were evaluated. Preliminary experiments show that the sensor can accurately identify the weights applied to its surface with a minimum detectable weight of 50 g within the sensing area and reflect the pressure exerted by weights ranging from 50 to 200 g in moving positions via conductivity images. The distinguishable number of the applied multi-touch weights was up to seven, which is superior to the current EIT-based tactile sensors where only two to four touchpoints can be imaged. These results show that the proposed sensor is capable of obtaining more information about the external contacts from multi-touch identification, thus enabling the EIT-based flexible e-skin for more sophisticated HRIs.

As a prototype of the e-skin, the proposed sensor has some limitations. For example, the sensor has a housing made of resin, which is not stretchable and deformable enough. If flexible materials like PVDF were employed, then the sensor would achieve greater softness and adapt to surfaces with a larger curvature (like cosmetic prosthetics). At the current stage, it is difficult to identify objects with complex shapes because the complex boundaries of the objects introduce uncertainties in the disturbance of the electric field within the sensing area and thus further exacerbates the nonlinearity and ill-posed nature of the EIT inverse problem. The most direct solution is to acquire richer boundary voltage data via increasing the number of electrodes in the hardware. When using a 32-electrode adjacent stimulation mode, each frame can collect 928 datasets, which is about 4.5 times the amount obtained using 16 electrodes. This can effectively reduce the ill-posed nature of the inverse problem but imposes higher requirements on the sampling speed. In this case, it is difficult to use an STM32 core to accomplish the task; a faster FPGA core would be a solution more suitable for the hardware design. Moreover, introducing deep learning models in the image post-processing will be an alternative to benefit the accuracy of image reconstruction. This approach does not require optimizing the existing hardware, but due to the extra image processing and data calculation involved, the imaging speed would be decreased, damaging the real-time response of the sensor. Future work will focus on updating the material and structure of the sensor and optimizing the reconstruction algorithm to eliminate the artifacts to meet the needs of real e-skin.

## Figures and Tables

**Table 1:** Comparison of the presented sensor with the existing EIT-based tactile sensors.

Research	Sensor Materials	Maximum Detectable Number of Touchpoints
Nagakubo et al. [2]	Conductive rubber	2
Wu et al. [14]	Conductive film made from polyolefin and nano carbon black	2
Zhao et al. [15]	Silicone as skin, water as liquid conductor	2
Visentin et al. [13]	Medical-grade highly conductive textile	3
Soleimani et al. [16]	Latex membrane as soft skin, an ionic liquid domain	4
Chen et al. [23]	Hydrogel	4
This work	Hydrogel	7

## Data Availability

Data are contained within the article.
